# Cooking chicken at home: Common or recommended approaches to judge doneness may not assure sufficient inactivation of pathogens

**DOI:** 10.1371/journal.pone.0230928

**Published:** 2020-04-29

**Authors:** Solveig Langsrud, Oddvin Sørheim, Silje Elisabeth Skuland, Valérie Lengard Almli, Merete Rusås Jensen, Magnhild Seim Grøvlen, Øydis Ueland, Trond Møretrø

**Affiliations:** 1 Norwegian Institute of Food, Fisheries and Aquaculture Research, Nofima, Ås, Norway; 2 Consumption Research Norway (SIFO), Oslo Metropolitan University, Oslo, Norway; University of Campinas, BRAZIL

## Abstract

About one third of foodborne illness outbreaks in Europe are acquired in the home and eating undercooked poultry is among consumption practices associated with illness. The aim of this study was to investigate whether actual and recommended practices for monitoring chicken doneness are safe. Seventy-five European households from five European countries were interviewed and videoed while cooking chicken in their private kitchens, including young single men, families with infants/in pregnancy and elderly over seventy years. A cross-national web-survey collected cooking practices for chicken from 3969 households. In a laboratory kitchen, chicken breast fillets were injected with cocktails of *Salmonella* and *Campylobacter* and cooked to core temperatures between 55 and 70°C. Microbial survival in the core and surface of the meat were determined. In a parallel experiment, core colour, colour of juice and texture were recorded. Finally, a range of cooking thermometers from the consumer market were evaluated. The field study identified nine practical approaches for deciding if the chicken was properly cooked. Among these, checking the colour of the meat was commonly used and perceived as a way of mitigating risks among the consumers. Meanwhile, chicken was perceived as hedonically vulnerable to long cooking time. The quantitative survey revealed that households prevalently check cooking status from the inside colour (49.6%) and/or inside texture (39.2%) of the meat. Young men rely more often on the outside colour of the meat (34.7%) and less often on the juices (16.5%) than the elderly (>65 years old; 25.8% and 24.6%, respectively). The lab study showed that colour change of chicken meat happened below 60°C, corresponding to less than 3 log reduction of *Salmonella* and *Campylobacter*. At a core temperature of 70°C, pathogens survived on the fillet surface not in contact with the frying pan. No correlation between meat texture and microbial inactivation was found. A minority of respondents used a food thermometer, and a challenge with cooking thermometers for home use was long response time. In conclusion, the recommendations from the authorities on monitoring doneness of chicken and current consumer practices do not ensure reduction of pathogens to safe levels. For the domestic cook, determining doneness is both a question of avoiding potential harm and achieving a pleasurable meal. It is discussed how lack of an easy “rule-of-thumb” or tools to check safe cooking at consumer level, as well as national differences in contamination levels, food culture and economy make it difficult to develop international recommendations that are both safe and easily implemented.

## Introduction

It is an increasing trend to eat poultry meat as a sustainable and convenient source of protein not associated with the negative health issues reported for red meat. At the same time, poultry meat is associated with the two pathogens ranked highest for health burden from food in Europe, *Campylobacter* and *Salmonella* [1].

A meta-analysis of 71 studies identifying risk factors for sporadic salmonellosis infections showed that amongst other factors, eating undercooked eggs or eating poultry at a restaurant were associated with salmonellosis [2]. More recent studies from Germany [3] and Australia [4, 5] also link sporadic cases of salmonellosis to poultry consumption. A meta-analysis including 72 studies identifying risk factors for sporadic campylobacteriosis infections [6] showed association with eating undercooked chicken and poor kitchen hygiene.

The contribution of the domestic setting to food borne illness is probably underestimated, but still about one third of the reported outbreaks in Europe occurred in the home setting in 2017 [7]. Among these, *Salmonella* was the dominating causative agent (various foods, most frequently associated with eggs) followed by histamine (mostly associated with fish) and *Campylobacter* (mostly associated with poultry). *Salmonella* is introduced to European households through poultry meat occasionally (4.9% of broiler samples are positive) and *Campylobacter* frequently (37.5% of broiler samples positive) [7] and the safety at the consumer stage relies on kitchen practices. It is generally recognised that during preparation of chicken in the domestic environment, undercooking, poor hygiene or a combination of both can potentially lead to campylobacteriosis or salmonellosis. Undercooking was reported in three out of eight domestically acquired outbreaks with *Salmonella* from broiler meat in 2017. The practices associated with illness were not identified in the remaining *Salmonella* outbreaks and the nine *Campylobacter* domestic poultry outbreaks [7].

The wide range of chicken recipes and products makes it difficult to develop standardised, safe cooking time-temperature recommendations for chicken preparation in the home [8]. Furthermore, the consumption pattern of chicken varies significantly between European countries, including differences in procurement process, type of chicken products and cooking styles [9]. As an alternative, safety can be built on proper ways of monitoring sufficient heat treatment. The recommendations for how to monitor chicken doneness vary between different authorities and other risk communication actors. Many actors mention that the meat juices should be clear and recommend a heat treatment to a minimum core temperature of 70°C. The European Food Information Council (EUFIC) recommends on their web page: “For pork and poultry, there should be no pink meat left. If you don’t have a thermometer, pierce the thickest part with a fork or skewer; the juices should run clear, not pink” [10]. A combined time-temperature regime (72°C for at least 2 min) is suggested for consumers who use a thermometer. The USDA [11] is advising on different minimum core temperatures for various foods, and at least 73.4°C for poultry. Discrepancies between the temperature recommendations can be explained by differences in food safety objectives and some variation in literature data on initial levels of pathogens and inactivation kinetics. Using a hypothetical Food Safety Objective for Salmonella in Poultry (up to 1 cfu/25 gram), JM Membre et al (2007) calculated that the performance objective for cooking should be set at 5.58 log reduction, corresponding to 0.25–0.43 minutes at 70°C depending on the approaches and assumptions [12]. Overall, it seems like a common view among experts that using a food thermometer should be the primary consumer advice for monitoring if poultry has been adequately cooked [10, 13]. For a number of reasons, the practice is infrequently adopted by consumers [14] and it has been argued that even a correct measurement of the core meat temperature is not enough to ensure safety [15]. However, even more questionable are the consumer recommendations related to meat colour and juices, since, as far as we know, these are not grounded in scientific evidence.

It is unclear what the present consumer practices are for deciding when chicken is ready cooked and if these practices are safe. A couple of studies from US indicate that undercooking (as defined by core temperature recommended by the authorities) is not a rare event. A combined observational and self-reporting investigation of consumer preparation of chicken breasts in the US in 2013 showed that appearance was the most common method for monitoring doneness. Consumers said they looked for “white coloured meat, absence of blood or pink spots and firm meat” [16]. About 40% of the participants in the study cooked their chicken to an unsafe final temperature (non-compliance with USDA recommendations, <74°C) and the author questioned if colour is an adequate indicator of doneness. Another US observation study showed that 24% of the consumers undercooked chicken fillets. A relatively high occurrence of use of thermometers were reported (37%) and several different monitoring methods, often used in combination, were observed, such as inner and surface colour, cooking time, juices, smell and texture [17]. In an observational study of Austrian consumers, outer colour was the most common method (78%), followed by internal colour (28%) and taste (10%) [18]. The safety of these methods was not evaluated. These few and scattered studies show some dissimilarities, but a large variety of approaches seems to be used to judge doneness by consumers and looking at colour seems to be much more common than using thermometers.

The aim of this work was to investigate how European consumers consider chicken meat to be ready for consumption This study thus employed a transdisciplinary approach, combining natural and social science, in order to investigate whether consumer practices and the current advice for monitoring chicken doneness are safe [19, 20]. The results indicate that advice from experts is not fully adopted by consumers. Furthermore, neither the recommendations nor the present consumer practices, will ensure sufficient inactivation of pathogens if the chicken is heavily contaminated with *Salmonella* or *Campylobacter*. Future food safety messages or tools should both ensure adequate heat treatment and take into consideration that consumer practices are habitual and motivated by other needs than safety.

## Materials and methods

The transdisciplinary research design in this study included three methodological approaches; qualitative consumer observations, cross-national quantitative consumer survey, and laboratory testing of chicken and food thermometers. In transdisciplinary studies on food safety, the collaboration of natural and social scientists has proven fruitful to produce positive outcomes for public health [19, 20]. In the present study, the combination of microbiology and sociology emphasized that critical food handling is a part of food cultures and thus varying within and across national borders. Qualitative consumer observation

### Consumer video-assisted cooking interviews

A transdisciplinary approach was employed for investigating the food safety when preparing a meal at home. Researchers from the same country, but representing different disciplines, a sociologist and microbiologist, visited a total of 75 European households in five countries (France, Norway, Portugal, Romania, UK) to interview and video film how consumers handle chicken and judge readiness of the meat. The research participants were instructed to prepare a meal of chicken and raw vegetables the way they would normally do it. The aim was to obtain an in-depth and detailed understanding of the ways consumers evaluate doneness including how they do it in practice, what they look for or what their aims are when deciding whether the chicken is ready to eat or needs more cooking, and, finally, how attitudes on food safety (if any) influence their cooking practices.

### Recruitment

The interviews and observations were part of a larger study where consumers were followed from shopping to consumption of food in their own home in five European countries; France, Norway, Portugal, Romania and the United Kingdom. Chicken consumption varies between these selected countries, including eating pattern, cooking repertoire, procurement and food traditions. While chicken has recently become a dominant food in the eating patterns of Norwegian and British consumers [21, 22] chicken has been influential in Romanian, Portuguese and French food cultures [23–25]. Three consumer groups were recruited; young single men (aged 20–29, living alone or flatmate, but not with a partner), families with infants/in pregnancy (couples or single parents, pregnant or youngest child aged 12 months or younger) and elderly (70 years or older) (S1 Fig). These three consumer groups were expected to differ in terms of vulnerability to food borne illnesses, familiarity to food safety messages and cooking routines and skills. All participants answered a screening questionnaire, including questions about their food habits of chicken and vegetables. In order to obtain a varied sample with regard to resources and challenges, a second set of recruitment criteria was employed including participants living in urban and rural areas with different income and education levels, and with either poor or adequate kitchen facilities and access to food stores. All participants were informed about research objectives, methodology, anonymization and that they could withdraw from the research process at any time, both verbally and by written information prior to the visits. All consumers signed an informed consent form. A recruitment agency, Norstat, was engaged to recruit all the research participants. Ethical approvals for the work were given by the Norwegian Centre for Research Data (Norway, 55256/3/AMS), The Ethical Panel at Keele university (UK, ERP1351), The National Data Protection Commission (Portugal, 13914/ 2017), The Ethical commission of University Dunarea de Jos (Romania, RCF1548/31.08.2017) and the Commission Nationale de l'Informatique et des Libertés (France, 152182 REC 0717 T001).

### Transdisciplinary working model

A transdisciplinary working model for the fieldwork observations and interviews was developed and piloted in 15 households in all the five countries, including an equal share of the three consumer groups. The working model provided instructions on what to observe and interview about, what to sample and also how to video-record the meetings/visits with the informants, including how to observe, when to ask questions as well as instruction of use of digital recorder, photo, video camera and equipment for sampling. The working model applied a shared conceptual model for studying food risk, integrates HACCP and practice theory. This meant that the primary focus was to observe the procedural steps of food preparation where risk could increase or decrease and to focus the interview on the practicality of cooking. As a result of the HACCP analysis, the cooking process was identified as a critical control point. Other parts of the process (e.g. those directly related to cross-contamination) are outside the scope of this paper. Questions asked by the researchers were careful and open, addressing the cooking only and avoiding moral ambiguities. When the participants finished cooking the meal, questions about food safety concerns were asked.

### Cross-national web-survey of consumer households

Complementary to the observational data, a consumer survey was conducted in the selected five countries to allow the measurement of problematic food handling behaviour in a standardised, quantitative and cross-nationally comparable manner.

#### Survey questionnaire

The survey included socio-demographic questions, consumption frequency of meals prepared from raw chicken in the household, usual level of chicken doneness for consumption in the household, and strategies for checking doneness level (Table 1). The survey also included additional modules on motivations, hygiene, food handling and on other food categories which are not reported here.

**Table 1:** Question items on chicken usage and cooking practices.

Item	Answer alternatives
How often do you or other members of your household eat dishes at home that you prepare from raw chicken?^1^	1 to 3 times per month
	Once a week
	2 to 4 times per week
	5 to 6 times per week

	Once a day
	2 to 3 times per day
When you eat chicken fillets at home, how 'done' do you usually have them?	Less done: white outside, pinkish inside and very juicy
	Medium-well: white outside, white inside and juicy meat texture
	Well-done: with some brown colouring outside, white inside and firm meat texture
	Very well done: with much brown colouring outside, white inside and very firm meat texture
When you heat chicken, how do you know that it is done? (*Multiple answers possible*)	I check how it looks from the outside
	I cut through a piece and check how it looks on the inside
	I poke it or pierce it with a fork and check if has the right texture
	I can tell from the juices
	I use a thermometer
	I always use a fixed amount of time
	Other
	None of the above

^1^ Only respondents who consume chicken at least once a month were included, which is why no alternative “less than once a month or never” is shown.

#### Consumer recruitment and data collection

Recruitment was subcontracted to a professional survey provider administering a large consumer panel worldwide (SSI, now Dynata). In each country (France, Norway, Portugal, Romania, UK) the population sample consisted of private households selected by stratified random sampling based on the Nomenclature of Territorial Units for statistics level 2 (NUTS2) of the respective country [26] and the education level of the target respondent. The within-country stratum sample sizes were proportional to the corresponding population stratum sizes. In the present paper, only data from households who declared preparing meals from raw chicken at least once a month were included. This ranges from 609 households in Portugal to 916 in the UK and gives a pooled sample of 3969 households across the five countries. Respondents consisted of 50.5% males and ranged from 16 to 90 years old (mean: 45.7 years). A bias towards higher education occurred as an artefact of running the survey online, with 55.1% of the respondents declaring a higher education. With regard to food safety, four risk groups of interest were represented in the sampled households: Pregnancy and children under six years of age; Diabetes and immuno-deficiency; Elderly above 65 years of age; and Young adults (teenagers, young adults and single men under 30 years old) leaving alone, with 55.4% of the households representing at least one of the four risk groups. Detailed socio-demographic characteristics of the respondents are presented in S1 Table.

#### Data preparation

The frequency consumption of dishes prepared from raw chicken at home (Table 1) was converted to days/month equivalents calculated by allocating proportional values to the original frequency categories with reference to a base value of 1.0, equivalent to once a month [27, 28]. The scores were calculated as follows: Monthly Frequency Equivalent (MFE) of 2 = 1 to 3 times per month, MFE of 4 = once a week, MFE of 12 = 2 to 4 times per week, MFE of 22 = 5 to 6 times per week, MFE of 30 = once a day, MFE of 60 = 2 to 3 times per day. Frequencies of self-reported practices are reported in terms of percentages per country and per age group.

### Laboratory cooking experiments

#### Chicken fillets

Chicken breast fillets (*Pectoralis major*) of 200–250 g used for determining inactivation kinetics for pathogens were purchased directly from a commercial Norwegian poultry slaughtering plant (Nortura, Hærland, Norway) on the day of slaughter (2018.03.20, 2018.04.16, 2018.04.24 and 2018.06.26), stored at 4°C and used for experiments within 2 days after slaughter. The breast fillets used for measuring colour, cooking loss and texture originated from the same slaughtering plant and were purchased from a grocery store, approximately 10 days after slaughter. At purchase, the fillets were packed in modified gas atmosphere (60% CO_2_/ 40% N_2_), 4–8 fillets in each package and each fillet was 150–170 g (fillets without tenderloin, *Pectoralis minor*).

#### Injection of fillets

Stock cultures were maintained in 20% glycerol at -80°C. Frozen suspensions of *Salmonella* were streaked on Tryptone Soy Agar (TSA; Oxoid, Basingsstoke, UK) and incubated at 37°C. *Campylobacter* was streaked on Mueller Hinton Agar (MH; Oxoid) and mCCDA (Oxoid) and incubated at microaerophilic conditions at 37°C (CampyGen CN0035A, Oxoid). Cultures for injection of poultry were grown in Brain Heart Infusion (BHI, *Salmonella*, 37°C, 24 hours) or Mueller Hinton Broth (MHB—*Campylobacter*, 37°C, 150 rpm, 2 days, microaerophilic conditions). Two inocula were prepared: The cultures (either 5 *Salmonella* strains or 6 *Campylobacter* strains) were mixed in equal volumes. The cocktails were diluted further in 0.9% NaCl to a final concentration of approx. 5 *10^7^ cfu/ml. The strains used in the experiments are shown in Table 2.

**Table 2:** Bacterial strains used in experiments. MF numbers refer to Nofima’s strain collection.

Bacterium	Source	Designation/name	Reference
*Campylobacter jejuni*	Poultry, Norway	C484, MF6842	[29]
*Campylobacter jejuni*	Poultry, Portugal	C21A, MF6883	Escola Superior de Biotecnologia (ESB) culture collection, Portugal
*Campylobacter jejuni*	Poultry, Denmark	DFVF1099, MF6903	[30]
*Campylobacter jejuni*	Turkey, Germany	C305, MF6901	[31]
*Campylobacter jejuni*	Human isolate	NCTC 11168, MF6902	
*Campylobacter coli*	Poultry, Portugal	C3, MF6878	ESB culture collection
*Salmonella* Typhimurium	Egg yolk, Portugal	SML1, MF6886	ESB culture collection
*Salmonella* Typhimurium	Eggshell Portugal	SML27C, MF6890	ESB culture collection
*Salmonella* Enteritidis	Human isolate Portugal	MF6974	ESB culture collection
*Salmonella* Senftenberg	Heat tolerant strain	775W; MF6898	[32]
*Salmonella* Infantis	Poultry, Hungary	M2016 ETBI 015346/01; MF6976	National Food chain Safety Office strain collection, Hungary

The chicken tenderloin was removed from all the fillets and fillets were injected with 5% v/w brine (e.g. 10 ml brine in 200 grams fillet) with or without the cocktail of pathogens using a syringe. The final concentration of pathogens was about 2*10^6^ cfu/gram. The whole volume of brine was distributed carefully by several injections (20–25 aliquots) by hand in each fillet to avoid leakage of meat juice and brine. The injected fillets were single packed in a vacuum pouch of polyamide/polyethylene (PA/PE) (Maske Gruppen, Sluppen, Norway) with an oxygen transmission rate of 50 cm^3^/m^2^, 24 h bar at 23°C and 75% relative humidity. Vacuum packaging was carried out on an Intevac IN30 chamber machine (Intevac Verpackungen, Wallenhorst, Germany) for the non-pathogen tests and a WEBOMATIC Computer 3000 S (WeboMatic, Bochum, Germany) for inactivation studies. The packages were not completely evacuated during vacuum packing, to minimise liquid loss and loss of inocula due to squeezing of the fillets.

The injected fillets were stored at 4°C for 16–26 hours before cooking. All injected fillets were weighed before injection to estimate correct volume of brine for each fillet. Fillets injected with brine without pathogens were also weighed after storage before cooking. About 2% liquid loss was found after vacuum packaging.

#### Cooking

The chicken fillets were cooked on two Silex S-161 plate grills (Elektrogeräte, GmbH, Arnsberg, Germany). The grill plate temperature was set at 165°C at the bottom plate and 180°C at the upper plate. There was a gap of approximately 5 mm between the top of the fillets and the upper grill plate. Soybean oil was spread on the dry bottom plate before grilling. The chicken fillets were cooked to core temperatures of 50, 55, 60, 65 and 70°C, respectively. The fillets were flipped after 10 minutes, and then flipped one or two times later, depending on the predetermined and measured core temperature. The core temperature during and after cooking of each fillet was recorded by a specially made laboratory thermometer, with separate probe type TKHånd (MRC Global, Skotselv, Norway) and a display box type Digitron 2000T (PSS Hire, Warrington, United Kingdom). The probe had a 1 mm thick needle thermistor and a fast response time, and the core temperature was measured by inserting the probe into the thickest part of the fillet in multiple spots when approaching final cooking. When the predetermined core temperature was measured as the lowest obtained temperature in the thickest part of fillet, the fillet was removed from the grill.

For the inactivation studies, three fillets per required core temperature were cooked each day and the experiment was repeated on three different days, resulting in a total of 12 fillets per core temperature. Chilled chicken fillets, with initial temperature of 4°C, were cooked three and three at the time, and placed on the plate one by one with 5 minutes intervals. When the predetermined core temperature was obtained, samples for the microbial analyses were taken immediately (less than 20 seconds after reaching the predetermined endpoint temperature). The temperature of the core was controlled also right after cutting, using an infrared thermometer (Raytek Raynger MX, Raytek, Berlin, Germany) and showed that it was not higher than the endpoint temperature. The samples were put into stomacher bags and placed in a refrigerator room (4°C).

Chicken fillets for colour and cooking loss studies were cooked three and three on the frying plate. In total, eight fillets of each core temperature were cooked and analysed. Chicken fillets used for texture analysis were cooked to core temperatures of 55°C and 70°C, eight replicates of each in total.

All chicken fillets were weighted before cooking and immediately after the predetermined core temperature was reached. Cooking loss based on weight (gram) was determined.

#### Microbial analysis

Core meat samples (3cm x 3cm x 1cm) were cut out and diluted 1:10 in sterile peptone water (*Salmonella*) or MH (*Campylobacter*). Samples were homogenized for 1 min with a stomacher (AES Smasher, AES Chemunex, Bruz, France). The homogenate was manually plated on PCA, XLD (Oxoid CM0469) and mCCDA. In one initial experiment the samples were also plated on MH agar and TS agar. If necessary, serial dilution in sterile peptone water or MH broth were spiral plated using a Whitley Automatic Spiral Plater (Don Whitley Scientific Ltd., West Yorkshire, UK). Incubation at 25°C for total viable counts on PCA for 72 hours, XLD for *Salmonella* counts at 37°C for 24 hours and mCCDA for *Campylobacter* counts at 37°C, microaerophilic conditions, for 72 hours. Fillet surface was swabbed 3x3cm (5x5cm for samples taken on day of injection) directly after cooking with FLOQSwab (Copan Flock Technologies, Italy), put in 3ml of sterile peptone water (*Salmonella*) or MHB (*Campylobacter*) and vortexed and plated as described for homogenate.

#### Sequencing colonies

Ninety colonies from three uncooked control samples and 90 colonies from three samples cooked until core temperature at 65°C (*Salmonella*) or at 60°C (*Campylobacter*) were sequenced on Genetic Analyzer 3500 (Applied Biosystems, Thermo Fisher Scientific, Waltham, Massachusetts, USA). Single colonies were added to 50 μl 1xTris-EDTA, lysed for 10 min at 99°C, centrifuged at 4000xg for 5 min and 30 μL supernatant was transferred and used as template. Amplification of the *hisD* gene for *Salmonella* using *hisD* forward primer (5’-GAAACGTTCCATTCCGC-3’) and *hisD* reverse primer (5’-CTGAACGGTCATCCGTT-3’). A 25μl-PCR reaction contained 10μl Platinum Hot Start PCR 2x Master mix (Invitrogen, Thermo Fisher Scientific), 0.2μM of each primer and 1μl DNA. PCR amplification was done by an initial step of 94°C for 2 min, 30 cycles x (94°C for 30 sec, 55°C for 30 sec, 72°C for 1 min) and a final extension for 7 min. Purification of PCR products was performed using 2μl ExoSapIT (Thermo Fisher Scientific) and 5μl PCR product with a thermal profile of 37°C for 30 min and 80°C for 15 min. A 20μl-sequencing reaction contained 3μl BigDye seq. buffer, 2μl BigDye Terminator v1.1 (Applied Biosystems), 2μl purified PCR product and 2μl 3.2μM *hisD* sequencing primer (5’-GTCGGTCTGTATATTCC-3’) was carried out using 25 cycles of 96°C for 15 sec and 60°C for 4 min. Final purification with BigDye X-Terminator Purification Kit (Applied Biosystems) as recommended from manufacturer before sequencing. Amplification of the *gltA* gene for *Campylobacter* colonies using *gltA* forward primer (A1 = 5’-GGGCTTGACTTCTACAGCTACTTG-3’) and *gltA* reverse primer (A2 = 5’-CCAAATAAAGTTGTCTTGGACGG-3’ as described for *hisD* gene except for a different amplification profile. *gltA* amplification was performed with an initial step of 94°C for 2 min, 35 cycles x (94°C for 30sec, 50°C for 1 min, 72°C for 1 min) and a final extension for 7 min. Sequencing as for *hisD* using 2μl 3.2μM *gltA* sequencing primer (S6 5’-CCAAAGCGCACCAATACCTG-3’) instead of *hisD* sequencing primer. We made use of the *Campylobacter* Multi Locus Sequence Typing website (https://pubmlst.org/campylobacter/) sited at the University of Oxford [33].

#### Colour analysis

Colour analyses of the chicken fillets were performed with a Minolta Chromameter CR-400 (Minolta Konica Sensing Inc., Osaka, Japan) with an 8 mm viewing port and illuminant D_65_. The instrument was calibrated against a white ceramic tile (L* = 97.16, a* = 0.25 and b* = 2.09). The cooked chicken fillets were sliced horizontally in the centre with a thin knife blade, and colour was measured in the middle of the thickest part. Three spots of each fillet were measured, which were averaged before further analysis. The fillet surfaces were covered with a thin wrapping PVC film to protect the instrument from vapour. Colour measurements were performed immediately after slicing. Colour measurements of raw chicken fillets were performed on the outer surface before cooking and were done on four fillets and at three spots on each fillet. The colour model decided by the International Commission on Illumination (CIE) for measuring colours, CIE L*a*b* (lightness (L*), redness (a*), yellowness (b*), was used to measure the colour of both cooked and raw chicken fillets. L* (luminance) has a value between 0 (black) and 100 (white). The a* describes the colour between red (a*≈120) and green (a*≈-120), and the b* value describes colours from yellow (b*≈120) to blue (b*≈-120).

#### Texture analysis

The meat texture was measured by monitoring the peak shear force, that means the highest recorded force in the Warner-Bratzler deformation curve needed to cut/split a piece of cooked meat. The fillets cooked to 55 and 70°C core temperatures were vacuum-packed, chilled over night at 4°C, and then conditioned at 20°C for 1 hour. Meat pieces of 1 x 1 x 2 cm were sliced along the fibre direction of the fillets. The pieces were cut across/perpendicular to the fibre direction with a Warner-Bratzler triangular device and measured for peak shear force with an Instron Universal Testing Machine type 5944 (Instron, Norwood, MA, USA). The analysis constituted of 8 fillets per temperature and 2 meat pieces per fillet.

### Evaluation of consumer thermometers

Eight different food thermometers were tested for accuracy, response time and practical features. All the thermometers were purchased in Norway, in-store or on-line, and the price of the thermometers varied between 5 and 200 Euros (Table 7). Similar thermometers are accessible worldwide. Five of the thermometers were intended for the domestic market. Two thermometers were more expensive and primarily marketed towards the commercial/industrial market (e.g chefs, industry) and the last thermometer was an expensive professional laboratory equipment with high accuracy and fast response time. The test constituted of 3 units of each type of thermometer, and each unit was tested 3 times. The accuracy according to target temperatures and response time was recorded at 3 designated temperatures; water with ice at 0°C, laboratory water bath at 70°C and boiling water at 100°C. The thickness of the probes was measured.

### Calculations

Chi-square and Cochran’s Q tests were conducted on chicken consumption and cooking practices variables to highlight significant differences within and across countries, risk groups or age groups at a 5% level. Pearson's chi-square test compares frequencies in one or more categories of a contingency table [34]. Cochran’s Q test handles multi-response frequency variables [35]. XLSTAT 2019.1.2 (Addinsoft, www.xlstat.com) was used for the calculations.

Minitab (Minitab 18.1, 2017, ww.minitab.com) was used to calculate mean values and standard error of the mean in the laboratory experiments.

## Results and discussion

### Consumer cooking of chicken: Qualitative results

#### Cooking processes

Most of the participants (39/75) prepared chicken fillets compared to whole chicken (19/75) and cuts of chicken (18/75) (Table 3). The type of chicken product prepared varied between consumer groups and countries. In Romania and France, young male participants typically prepared chicken fillets, while in Portugal fillets were typically prepared by families with infants and pregnant women. In Norway and the UK, chicken fillets were prepared equally among all the consumer groups. Whole chicken was mostly prepared by the French and the Romanian participants. Cuts of chicken were more typical among the Portuguese participants. The results reflected differences among the countries with regards to the production and retail of chicken and food cultural traditions and preferences. Furthermore, they are also related to where the chicken is bought–from a butcher or from a supermarket shelf.

**Table 3:** Overview of chicken products prepared in the consumer fieldwork. The number of participants preparing a type of product divided by country and consumer group is shown.

	Portugal			Romania			France			UK			Norway			N
	YM**	YF	E	YM	YF	E	YM	YF	E	YM	YF	E	YM	YF	E	
**Whole chicken**	-	-	1	3	2	4	2	2	4	-	1	-	-	-	-	19
**Cuts (parts with bones)**	2	2	5	-	1	1	-	2	1	1	1	1	-	1	-	18
**Fillets (no bones)**	1	4	-	3	2	-	3	1	-	4	3	4	5	4	5	39
**N**	3	6	6	5	5	5	5	5	5	5	5	5	5	5	5	76*

*A Romanian young man prepared two types of chicken products.

** YM: Young man; YF: Young family; E: Elderly.

Several cooking methods were observed among the participants in the study. Methods such as frying and cooking the chicken in a pan or in the oven were most common. In addition, microwaving, using a cooking machine and scalding the chicken over the gas were observed among a handful of participants. Typically, fillets and cuts of chicken were heated on the stove (fried or boiled) while whole chickens were cooked in the oven indicating that the cooking method is associated with the type of chicken product.

#### Judging doneness

All the participants in this study cooked and ate a meal of chicken at least once every fortnight. Chicken was regarded as tasty, healthy, a good source of protein (typically among the Britons), convenient food for children (among the Norwegian families) and as traditional meat to eat (among the French, Portuguese and Romanian participants). The observations revealed that judging doneness is procedurally integrated to the cooking process since consumers are monitoring the chicken from the start to the end of the heating process. Two broad motives were expressed by the participants when judging if the chicken was done. Undercooked chicken was perceived by most informants as risky to eat. Meanwhile, participants also mentioned that chicken was especially vulnerable to long cooking time, making it dry and not very pleasant to eat. For some, cooking chicken was thus a question of heating it enough without losing the softness or juiciness of the meat. These two dimensions–safety and tastiness–may very well come in conflict. Meanwhile, they are important for interpreting how and why the participants decide if and when the chicken is properly cooked. Nine ways of determining doneness were identified (Table 4). Most of these practices have been reported also in other observation studies [16–18, 36], with the exception of using sound.

**Table 4:** Different ways of determining doneness among consumers. Consumer methods for determining doneness and the number of consumers where the practice was observed are given. The total number of households visited was 75.

Method of determining doneness	Number
Timing cooking based on experience	40
Looking at the colour of the surface of the chicken meat	34
Looking at the interior colour of the meat	33
Judging the texture of the chicken (using utensils)	30
Second heating processes (intended)	23
Using a recipe, following time and temperature instructions	8
Tasting the chicken meat	6
Frying sounds and smell	5
Using a thermometer	1
N (number of times methods observed)	150

*The identified methods are not exclusive. Some of the ways identified can be separated into sub-categories, others can be merged.

Most of the participants (61/75) made use of more than one method for deciding when the chicken was cooked enough to be served. The combinations of methods were many, and method(s) employed depended on the type of chicken product cooked and the heating method employed.

More than half of the participants (40/75) determined doneness by timing the cooking approximately, making use of their cooking experience and skills. A few participants told they had at some point used a recipe for the chicken dish they made but learned it by heart after cooking it several times. Timing the cooking approximately was a method for determining doneness regardless of the type of chicken product cooked. While some participants used a timer (on the oven or on the smartphone) or had an eye on the clock to measure time, others relied more on “sensing” time. Some participants timed the cooking of chicken with the help of the cooking time of other foods such as rice, potatoes and pasta. A Romanian single man aged 31, checked the doneness of the potatoes he cooked in the same pot with chicken to know when the chicken was properly cooked. A few participants, most of them French (8/75) told that they cooked chicken meat “as long as possible”. This was typically advocated among the French elderly participants who cooked a whole chicken in the oven. Those who fried chicken fillets, on the other hand, expressed that too long cooking time would lead to dry meat.

Quite a few consumers (34/75) monitored the heating process of the chicken by looking at the surface colour, for instance to avoid burning. Meanwhile, many voiced that the reason for doing it was to make sure that the chicken cooked properly, for instance, that all surfaces of the chicken meat were evenly cooked during frying. Checking the surface colour was also an important step to reach pleasant taste. For example, a French young man aged 25 expressed that he preferred chicken with a golden-brown surface colour and soft, juicy meat. He was one of few participants who only checked the outside colour before deciding that the chicken was properly cooked. For most of the participants, visual appearance of the surface browning was only the first step to assess the progress of the cooking, followed by either cutting the chicken to look at the inside colour of the meat or a second heating process (e.g. cooking it in a sauce, adding it to an oven dish or a casserole) when the chicken had achieved the desired colour.

Almost half of the participants (33/75), checked the colour of the meat to see if it was properly cooked by cutting into the meat with a knife, splitting it with a spoon, fork, tong or spatula, sometimes while still in the frying pan or ripping it in parts using one’s hands. Participants were looking for the pinkish colour, which meant that the chicken was still not properly cooked, while a white colour signalled that the chicken was ready to eat. For the participants cooking a whole chicken, blood was considered a sign of undercooked chicken. Checking the colour of the meat inside was most often done by the British participants, among all the households. In comparison, among the Norwegian participants, it was typically done by the young male participants. In the Romanian and French households, particularly the elderly participants checked the colour of the meat, while among the Portuguese, this was typically done among the young families.

Another common method employed to decide if the chicken was properly cooked was to check the texture of the chicken. Almost half of the participants used various ways and tools to feel the firmness or the consistence of the meat. It was possible to distinguish between two methods; 1) squeezing, poking or prodding the chicken, and, 2) pulling the chicken apart from its bones. The first method was more common among the participants who cooked chicken fillets than the second, which was common when cooking a whole chicken. Checking the texture by squeezing, poking, prodding, and splitting the meat using various utensils (e.g. spatula, tongs, knife, forks, spoons, fingers) was arguably a more subtle or unarticulated way of judging doneness. Few told explicitly why they pursued to poke or prod the chicken or articulated what they were sensing. Among the Norwegian participants “bounciness” or feeling how much the meat “gives in” were mentioned, but few provided any detailed descriptions. Thus, when frying the meat people receive sensory feedback about the changing physical state of the chicken in the process of moving it around, but in ways that they might not be able to articulate or even be conscious of. One 23-year-old British young man demonstrated that the pieces of chicken fillet became easier to break apart along the cooking process. For him, this was a telling sign of the cooking progress helping to determine when the chicken was properly cooked. Other unspoken or subtle sensory inputs were also observed, including smell and sound alerting the participants to do something (e.g. turning the chicken, checking if it was getting burned). Also, in Portugal, a young single man claimed that smell was a good indicator to check for chicken doneness. Only a few participants articulated the role of sound or smell, but these signals may still have been a part of the subtle and unspoken way of monitoring doneness when cooking chicken among others as well.

Many of the participants heated the chicken two or more times as part of preparing the meal. Almost a third of the participants (23/75) employed a second round of heating to the same chicken, often frying or searing the chicken first, followed by a boiling or stewing process. The second heating was more common among the Norwegian and British participants. A few explained that a second heating was necessary to ensure that the chicken was properly cooked. For example, a Norwegian mother aged 37 said she would not have eaten the chicken after frying it because it was undercooked, but she didn’t worry too much because the chicken would cook further in the coconut sauce she was preparing. Others mentioned the same in a less explicit way. A second cooking procedure was more common among those who heated the chicken on the stove (frying or cooking) than in the oven (roasting, microwaving, using a cooking machine). None of the participants who cooked a whole chicken heated the chicken a second time.

Using a recipe or any type of time and temperature instruction on how to cook chicken properly was not widespread among the research participants. Only eight of the 75 participants followed a recipe or instruction to determine how to cook the chicken enough. Similarly, few (7/75) tasted the meat to check if it was properly cooked. The participants did not taste the chicken to find out if it was raw. Instead, it seemed that they tasted it to check if the chicken was hedonically ready–if it had a pleasant taste—perhaps to avoid it from cooking too much.

Only one participant used a thermometer to determine if the chicken was properly cooked: an elderly Norwegian woman aged 70 years, who stated using a thermometer for all kinds of meat and cuts of meat, including diced chicken fillet. Other participants mentioned that they could use a thermometer for instance when roasting a whole chicken or turkey in the oven, but none of the participants cooking the chicken in the oven during our field work used a thermometer.

The practices observed were partly in accordance with what has been reported in other studies, with few consumers using food thermometers and relatively many using a visual judgement of the meat [16–18, 36]. A significant difference from other studies is the large fraction of people using “cooking time based on experience”. This may be a result of differences between study designs, both resulting from different scopes and interview techniques. A challenge with observational studies is not only that the subjects may act differently when being observed, but also that the observations may be difficult to report and interpret. In some studies, “cutting” or “visual inspection” are reported as methods of judging doneness, without any further explanation, and in other studies the scope is to determine whether observations are in compliance with certain standards (e.g. using a thermometer or not). Our study design used a combination of observation and interview techniques developed to make the research participants talk about their cooking activities, including less explicit mundane routines such as using “an inner clock” or an internalised cooking recipe. Cooking as a less reflective activity influenced by past experiences [37]and consumers’ confidence in their own ability to handle and prepare food safely at home has been identified in other studies [38]. Nevertheless, both from the present study and other studies, despite differences between countries, consumer groups and types of chicken products prepared, a range of criteria are used by consumers to judge doneness, and one criterium is seldom used alone.

### Consumer cooking of chicken: Quantitative results

Home consumption frequency of dishes prepared from raw chicken at home were on average of 7.6 days per month equivalents across the multinational sample. National averages ranged from 5.9 days/month in Norway to 9.8 days/month in Romania (Table 5). Among risk groups, young men were the most frequent preparers of raw chicken with 10.9 days/month equivalents, which is twice as frequent as the elderly (5.6 days/month equivalents). In line with this result, a global generational gradient was observed with a decrease in chicken consumption frequency with increasing age (S2 Table). In an overall perspective, households prevalently declared consuming chicken meat that is well-done (56.9%). Contrary to all other countries, fewer households in Portugal consumed very-well cooked chicken (8.4%) compared to medium-well cooked chicken (26.4%). A higher proportion of elderly tended to prefer chicken meat well/very well done than the young groups (86.8% vs 77.8%) (S2 Table). Among groups, 2.6% of young men and 1.7% of young families declared consuming chicken less done, indicating a higher risk behaviour on that aspect than those with fragile health (0.8%) and the elderly (0.1%) (Table 6).

**Table 5:** Self-reported practices per country.

Country	FRANCE (n = 706)	NORWAY (n = 844)	PORTUGAL (n = 609)	ROMANIA (n = 894)	UK (n = 916)	POOLED (n = 3969)
Raw chicken frequency*	6.2	5.9	7.5	9.8	8.1	7.6
**Doneness of chicken (%)**						
*Less done*	1.1^a^	0.7^a^	1.0^a^	1.1^a^	0.7^a^	0.9^a^
*Medium-well*	18.3^b^	16.4^b^	26.4^b^	12.4^b^	19.1^b^	18.0^b^
*Well-done*	56.2^c^	53.6^d^	64.2^c^	59.1^d^	53.6^d^	56.9^d^
*Very well done*	24.4^b^	29.4^c^	8.4^b^	27.4^c^	26.6^c^	24.2^c^
**Strategy for checking doneness (%)**						
*Outside colour*	38.2^d^	21.8^d^	35.0^d^	39.1^c^	21.5^b^	30.6^d^
*Inside colour*	39.1^d^	61.0^e^	42.0^e^	51.1^e^	50.8^e^	49.6^f^
*Inside texture*	42.4^d^	21.6^d^	44.8^d^	58.6^d^	30.1^c^	39.2^e^
*Juices*	11.6^b^	12.4b	15.4^b^	14.5^b^	37.8^d^	19.1^c^
*Thermometer*	5.9^a^	8.9^a^	3.4^a^	3.4^a^	11.0^a^	6.8^a^
*Time*	24.1^c^	11.5^c^	14.3^c^	12.9^b^	15.9^a^	15.5^b^

*Expressed in days/month equivalents.

^a,b, c, d, e,f^: Different letters indicate significant differences across alternatives within country (Cochran’s Q test).

**Table 6:** Self-reported practices for different consumer groups. The groups were young men (<30 years old), young families (expecting or with children), persons of fragile health (diabetes, immuno-deficiency), elderly (65+ years old) and other respondents (i.e. miscellaneous participants).

Risk group	Young men (n = 461)	Young families (n = 702)	Fragile health (n = 828)	Elderly (n = 761)	Other (n = 1770)
Raw chicken frequency*	**10.9**	**9.3**	**8.7**	**5.6**	**7.4**
**Doneness of chicken (%)**					
*Less done*	2.6	1.7	0.8	0.1	0.6
*Medium-well*	22.8	16.5	17.6	18.8	18.6
*Well-done*	49.0	55.8	56.4	57.6	57.5
*Very well done*	25.6	25.9	25.1	23.5	23.3
**Strategy for checking doneness (%)**					
*Outside colour*	34.7	30.3	28.3	25.8	31.9
*Inside colour*	40.6	44.0	45.2	46.9	52.8
*Inside texture*	31.7	34.3	44.9	46.1	36.8
*Juices*	16.5	17.2	20.0	24.6	17.4
*Thermometer*	10.6	9.1	8.6	6.3	6.0
*Time*	18.0	15.4	17.5	16.7	15.5

* Expressed in days/month equivalents.

Further, households most typically checked cooking status from the inside colour (49.6%) and/or texture (39.2%) of the meat. However generational differences were observed, where the younger age group (16–30 years old) relied more often on the outside colour of the meat (33.3%) and less often on the juices (15.2%) than the eldest age group (76–90 years old; 22.6% and 34.0%, respectively) (S2 Table). We have not identified other studies showing this result. However, a study comparing young and adult consumers and their preference for consuming meat in general at different levels of doneness show similar results [39]. Only 6.8% of our surveyed households declared using a cooking thermometer for chicken preparation, with a higher prevalence in the UK (11.0%) and in Norway (8.9%). It is noticeable that 25.8% of elderly, 28.3% of those with fragile health, 30.3% of young families and 34.7% of young men may interpret the outside colour to determine chicken doneness.

### Relation between core temperature, microbial inactivation, colour, texture and water loss

#### Cooking time

**Fig 1:**
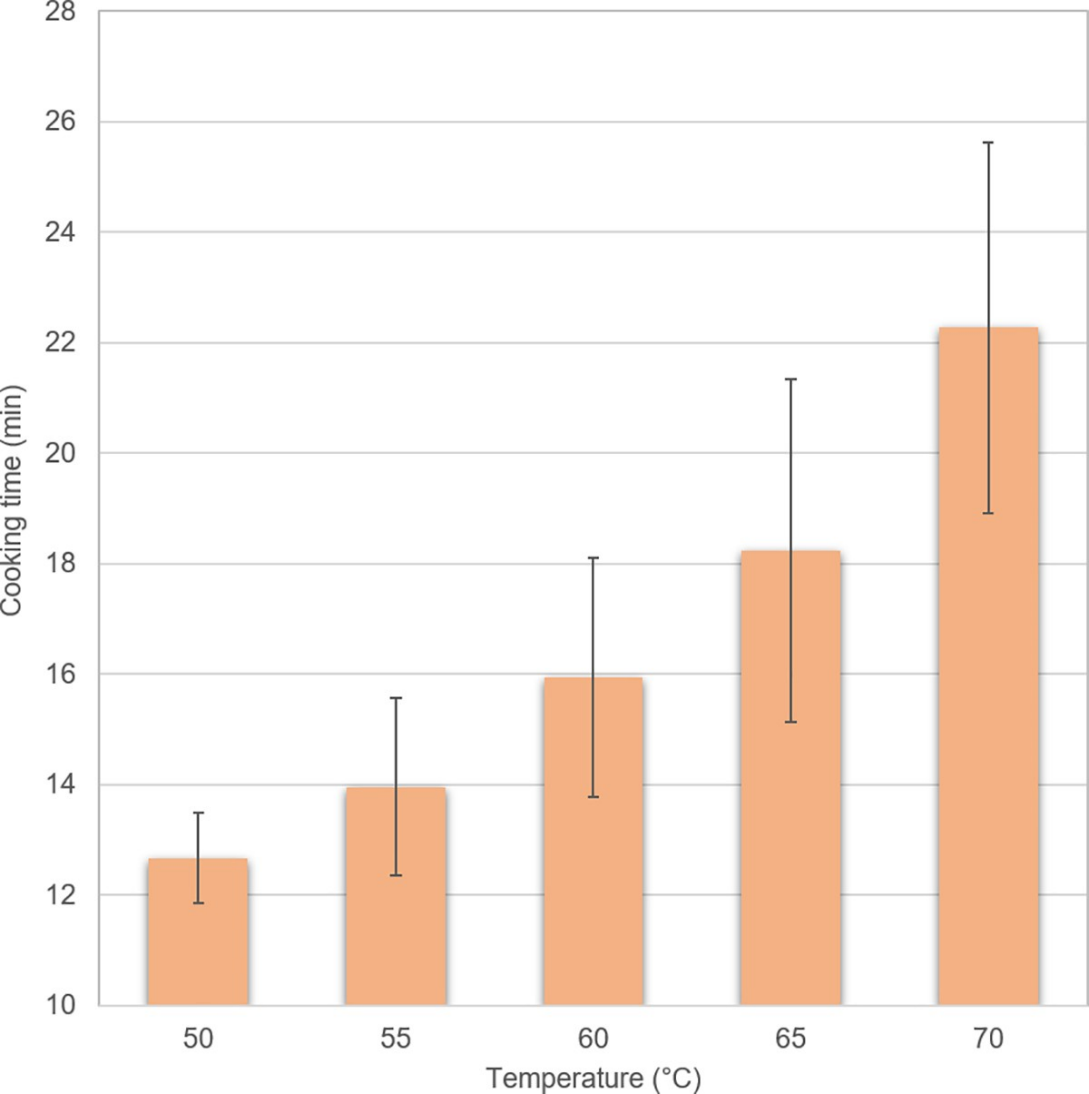
Cooking time of chicken fillets in minutes for the different end core temperatures. Mean values and standard error for 15–26 fillets are shown.

As found in the observation study, nearly half of the consumers used time (most based on experience, a few on a recipe) to determine whether the chicken was ready. The actual core temperature at the end of cooking was not measured in the field work, partly because it would potentially disturb the observations and partly it would be impossible to standardise the methodology to get comparable results. About 15% reported that they used a fixed time when preparing chicken in the survey. Pilot kitchen experiments showed that the cooking time to obtain a certain core temperature was highly variable (Fig 1). In these experiments, an experienced and trained technician cooked fillet with similar weights, used a strictly standardised method for frying and a tailormade food thermometer to measure temperature. Thus, the variability in the consumer setting is likely much higher indicating that defining a cooking time to obtain a consistent result would be difficult.

#### Inactivation of microbes

**Fig 2:**
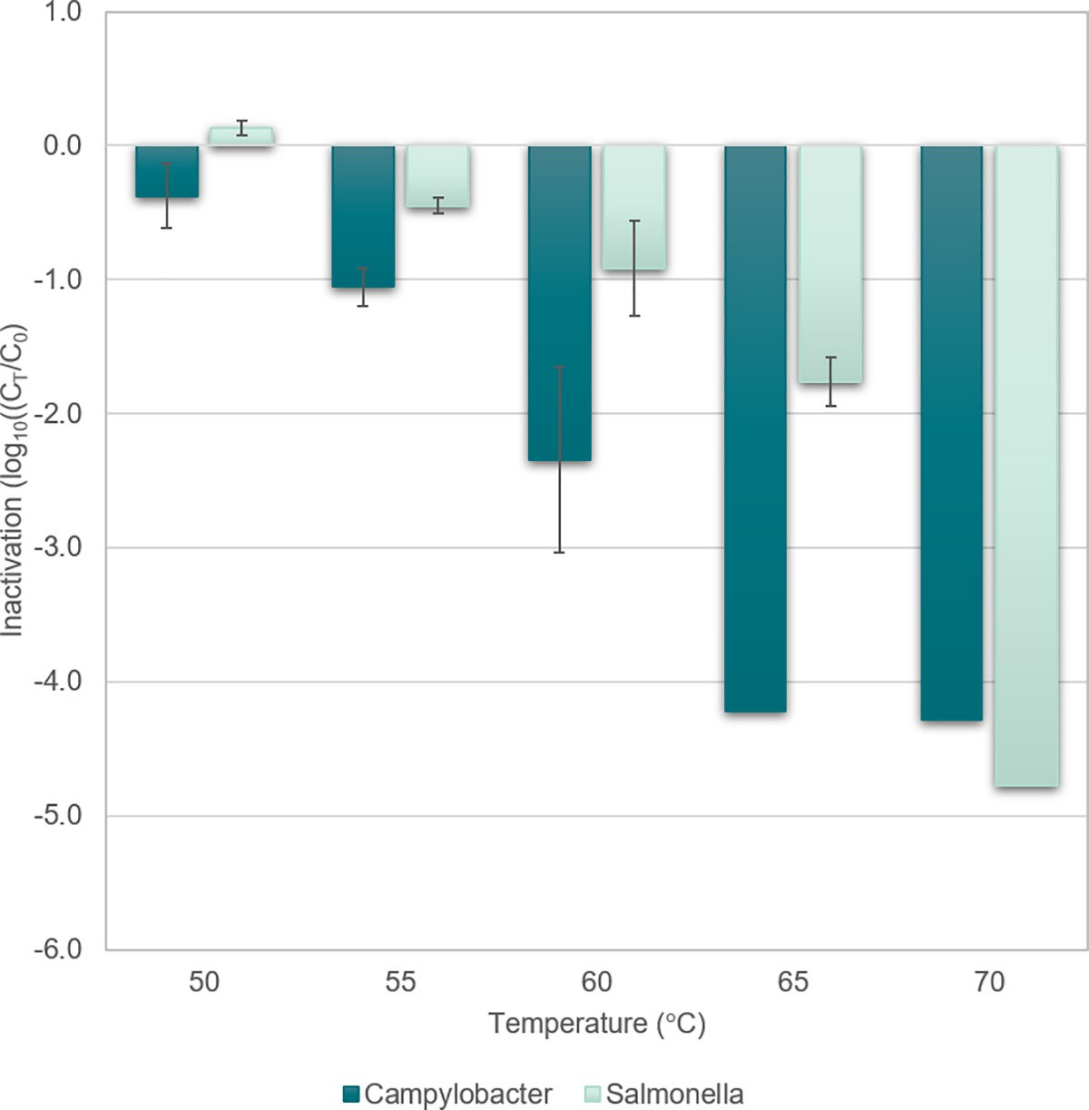
Inactivation of *Campylobacter* and *Salmonella* in the core of the fillets having reached different core temperatures. The inactivation was calculated as the ratio of viable organisms after the core reached a certain temperature (C_T_) and before cooking (Co). Mean and standard error for log transformed values are shown. Columns without error bars indicate numbers below the detection limit.

According to the consumer recommendations from WHO, food should be cooked until a core temperature of 70°C to be safe [40]. When the core temperature reached 70°C, the number of surviving bacteria in the core of the fillets were below the detection limit of the experiment, of at least 4 log reduction (Fig 2). The inactivation at different end temperatures were similar to what was reported in a comparable study mimicking consumer frying of poultry burger [41].

*Salmonella* spp. appeared to be more resistant to heat than *Campylobacter* spp. as about 2 log reduction was found at a core temperature of 65°C, while the reduction of *Campylobacter* was more than 4 log. Higher resistance of the former is not surprising as earlier studies in laboratory media have shown a decimal reduction time of 0.1–3.3 minutes for *Salmonella Enterica* and <0.01–0.11 minutes for *Campylobacter* spp. at 60°C [42]. Also, in a thermal inactivation model based on inactivation data from several food products and laboratory media, the decimal reduction time was higher for *Salmonella* than *Campylobacter* at temperatures below 85°C [43]. In contrast to *Campylobacter*, several studies have been conducted on inactivation of *Salmonella* in media based on poultry meat. While the D_70_ values are in range of seconds, D_65_-values vary between 0.5–1 minute and D_60_-values 4–8 minutes. Although these experiments are not possible to compare directly with the present study, the inactivation obtained seemed to be within the same range.

As the majority of the pathogenic contaminants of chicken fillets occurs on the surface and not the interior [44], inactivation of *Campylobacter* and *Salmonella* on the surface of the chicken fillet is crucial. For surfaces in contact with the frying plate, more than 4 log reduction was observed at core temperatures as low as 50°C and a frying time of 12 minutes. In accordance with this, more than 6 log reduction of *Campylobacter* on the chicken fillet surfaces in contact with the frying pan was obtained after a frying time of 12 minutes in another study [15].

**Fig 3:**
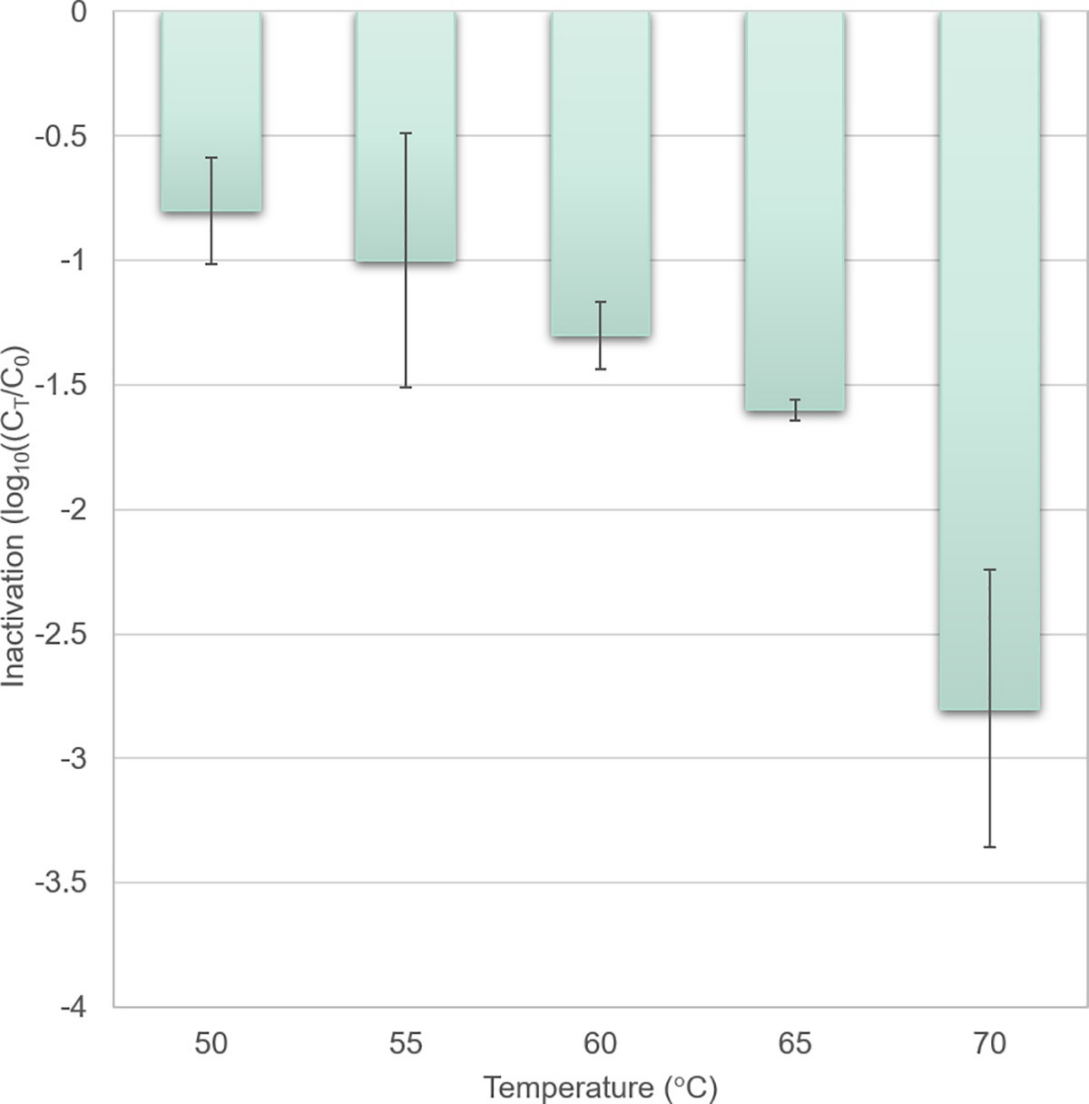
Inactivation of *Salmonella* on the surface of poultry fillet not in contact with the frying plate. The inactivation was calculated as the ratio of viable organisms after the core reached a certain temperature (C_T_) and before cooking (Co). Mean and standard error for log transformed values are shown.

As shown in Fig 3, at the surfaces that were not in contact with the frying plate, survival was observed, even when the core temperature of the fillets reached 70°C. The inactivation of *Campylobacter* was similar to what was found for *Salmonella*. Mean inactivation rates were not possible to calculate for *Campylobacter* as the logarithmic reduction at 65 and 70°C varied from 2 to > 3.5 (detection limit). Depending on the thickness of the fillets, it was noticed that the meat surfaces sampled after the core temperature reached 65–70°C, sometimes looked undercooked (pink and glossy surface).

**Fig 4:**
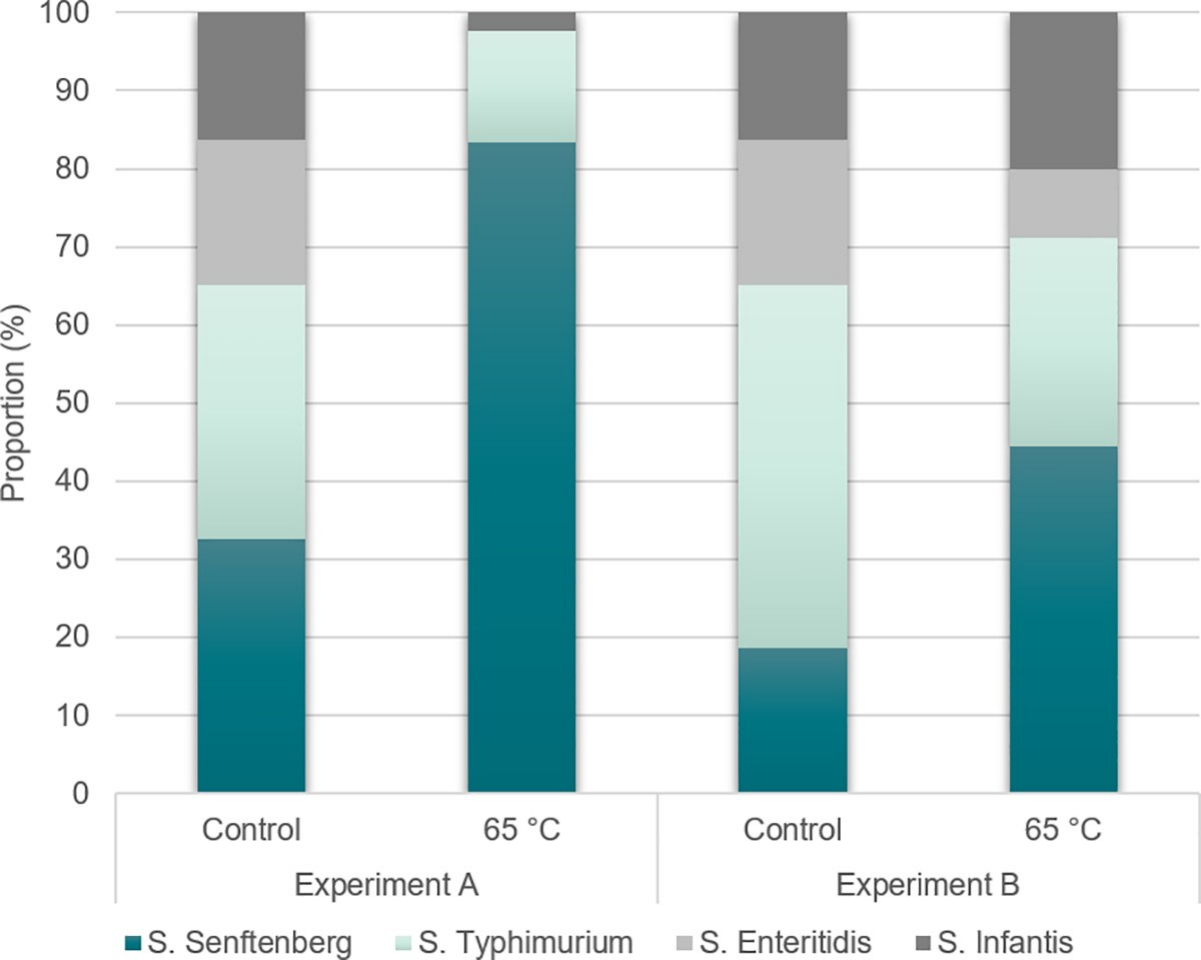
Relative proportion (%) of *Salmonella* strains before (control) and after cooking to a core temperature of 65°C. Results from two experiments (total 90 sequences per condition from both experiment) is shown. The data for *S*. Typhimurium MF6886 and 6890 are shown together, as the strains could not be separated in the analysis.

*Salmonella* Senftenberg 775W was included among the strains in the study because it has been reported as highly resistant to heat [45]. Sequencing of isolates from uncooked and cooked samples showed a tendency that *Salmonella* Senftenberg 775W dominated after, but not before cooking (Fig 4), indicating that differences in heat tolerance may have practical implications. Thus, in most cases, where chicken is contaminated by other Salmonella serotypes, the inactivation during consumer heat treatment of Salmonella will be higher than in the present study. No selection of specific strains of *Campylobacter* after cooking was found.

From the inactivation experiments, it seems like cooking to an internal temperature of 70°C will inactivate (>5 log reduction) pathogens in the interior, but not necessarily on the surface of the chicken fillets not in contact with the frying plate (< 3 log reduction). In most cases, the levels of pathogens in positive samples of chicken at the retail level are low. However, a small fraction of chicken carcasses containing more than 10^5^ and 10^4^ cfu/gram of *Campylobacter* and *Salmonella* respectively has been reported [46, 47]. The majority of pathogens are present on the surface. As an example, Luber et al [44] found up to 40 000 cfu *Campylobacter* on the surface of German chicken fillets but maximum 100 cfu in the interior. Thus, from our results, it seems like cooking to an internal temperature of 70°C will eliminate pathogens to safe levels in the interior (> 5 log reduction) in most cases, but not necessarily on the exterior (< 3 log reduction).

### Colour and texture changes during cooking

**Fig 5:**
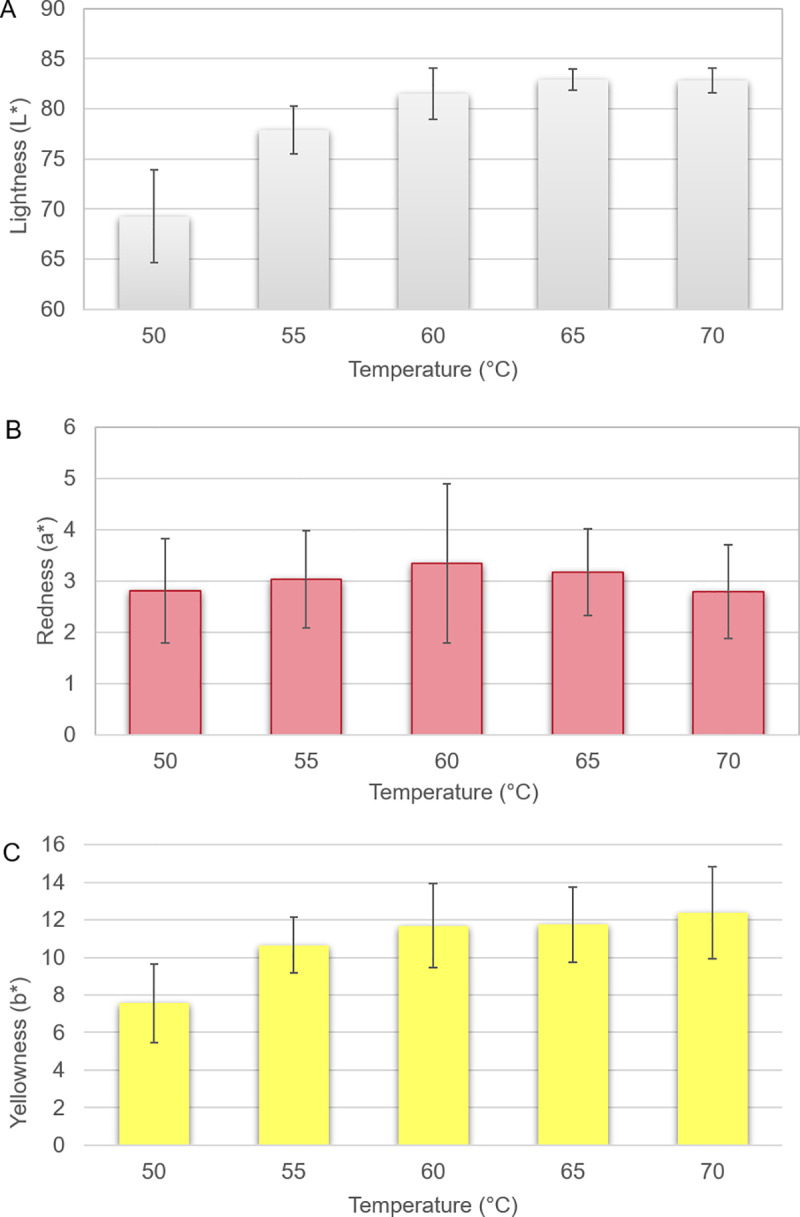
Lightness, redness and yellowness of core meat after cooking to different core temperatures. Mean L*, a* and b* values and error of the mean shown.

**Fig 6:**
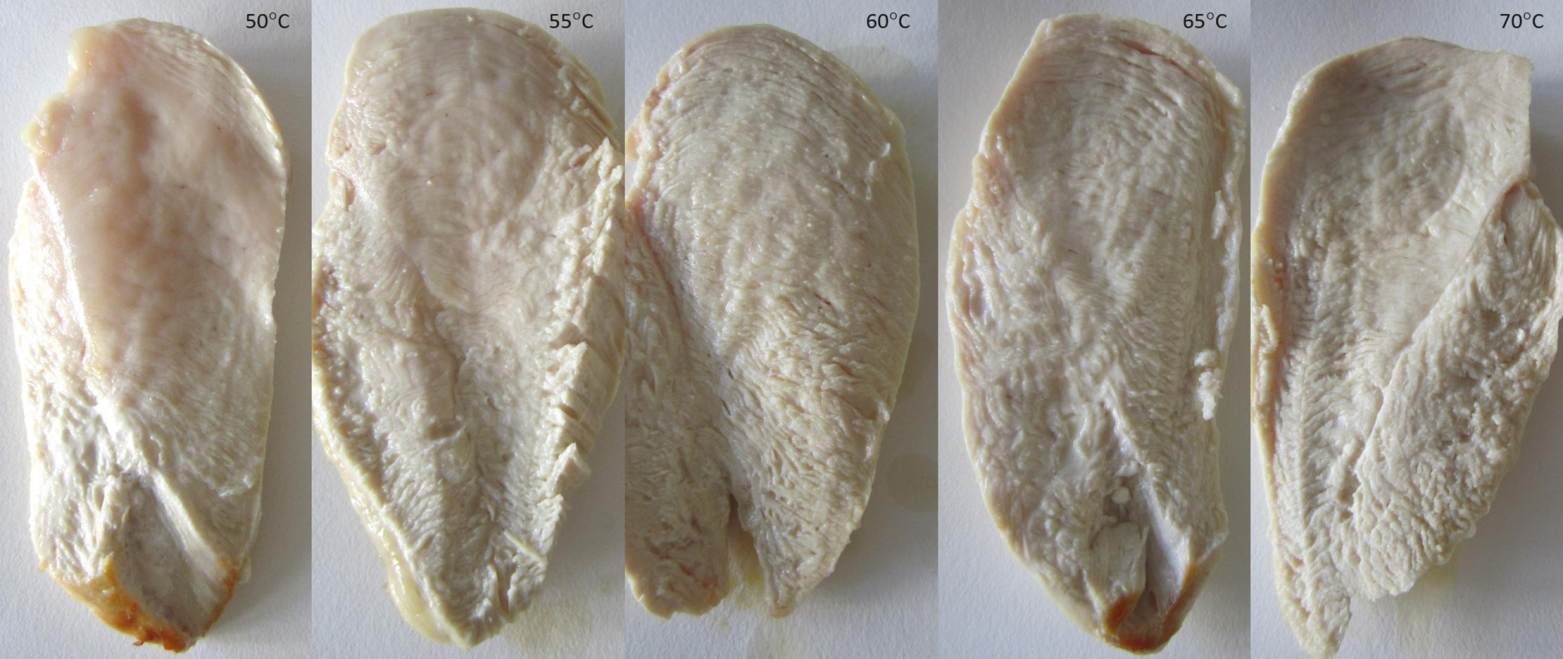
Pictures of fillets at different end temperatures (50–70°C).

Some food safety risk communicators mention colour of the core of the chicken meat or the juices as signals for doneness as alternatives to using a food thermometer [10, 48]. Checking the interior colour was also done by about one third of our informants in the observation study and reported by almost half of the respondents in the survey. Instrumental analysis showed that most of the colour change during cooking happened before the core reached 55°C (Figs 5 and 6). Significant differences were obtained for L* (lightness) between 50 and 55, as well as 55 and 65°C or higher, and for b* (yellowness) between 50 and 55°C (P<0.05). For a* (redness), no significant differences between temperatures were recorded (P>0.05). Raw chicken fillets are pale and low in content of the myoglobin pigment and increasing degree of cooking did not reflect change in colour for the critical temperature for food safety near 70°C. The raw fillets had a L* value of 55.4 ± 2.8 a* value of 2.6 ± 0.9 and b*value of 4.6 ± 2.1.

About one fifth of the consumers reported that they check the colour of juices from the chicken to see if it is done. The colour of the meat juice at temperatures between 50 and 70°C was too pale to enable detection by the instrument.

Most studies on the colour of chicken during cooking are primarily focused on the pink colour that may develop at temperatures above 70°C (see example Bae et al and references therein [49]). Surprisingly, studies on the relation between colour of chicken fillets and core temperature during cooking are almost lacking. Rabeler et al (2019), reported about colour change (increasing lightness) over time during convection cooking of chicken fillets [50]. As corresponding core temperatures were not measured, it is difficult to compare with our results. In another study, they modelled colour changes during thermal treatment using a thin slices of chicken meat cooked in water [51]. In their model system, lightness increased rapidly, and redness declined at temperatures of 65°C and above, while at 50°C, the changes were slow and never reached the same values. In contrast to this, in our study, the a*-values were low even at 50°C and did not change by cooking to higher temperatures (75°C were included in initial experiments and resulted in the same colour profile as 60°C). Differences in results could be a result of dissimilar cooking model systems, but also variations of raw materials since the colour of chicken fillets depend on environmental and genetic factors [52]

As shown in Table 4, some consumers used meat texture, using a utensil or fingers as indicators of doneness. The meat texture at different temperatures was measured, and the maximum shear peak force of the cooked meat was 9.8 ± 2.4 and 12.5 ± 1.7 N at 55 and 70°C core temperature, respectively. The two temperatures represent conditions where the myofibrillar protein is mostly undenatured and denatured. The results on peak force at 55 and 70°C are so similar that it is unlikely that most consumers will be able to distinguish between safe and unsafe cooking of chicken breast fillets based on texture. In contrast to our finding, Barbanti & Pasquini (2005) found a trend of higher shear force values with increasing heat treatment of chicken fillets [53], but apparently with a larger span between the lowest and highest cooking procedures than in our study.

Photos of the inner part of cooked fillets at various core temperatures are shown in Fig 6. At 55–60°C the meat has a dense and glossy appearance. However, by increasing the temperature to 65–70°C, the meat changed to exhibit a coarse fibre structure. Development of a fibrous structure and loss of glossiness of the fillets, which probably reflected the initiation of protein denaturation, were more profound than the colour change between 60 and 70°C. About 40% reported that they checked inner texture in the survey (Table 5), but details about how this was performed were not given.

### Cooking loss

**Fig 7:**
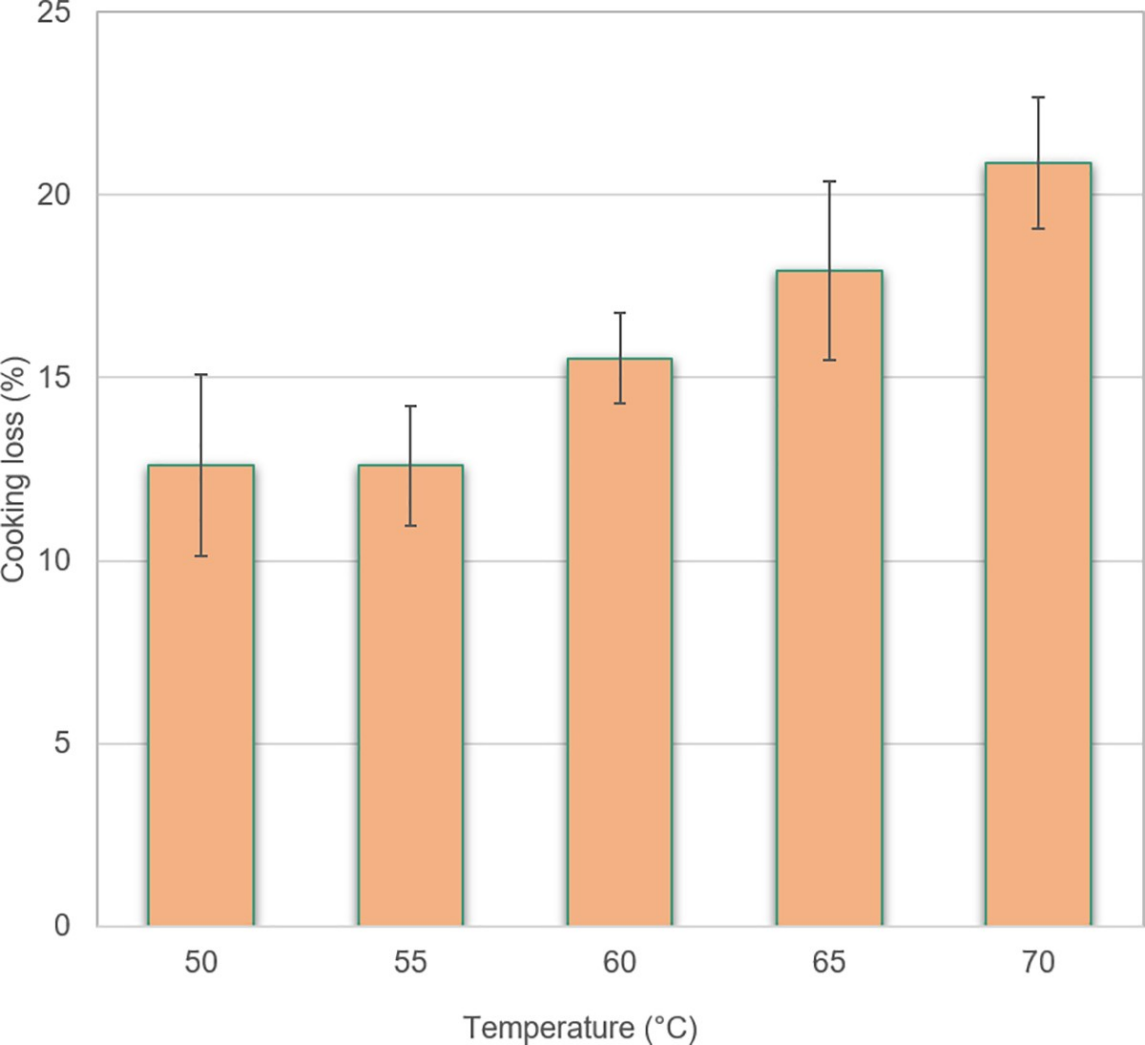
Cooking loss (%) of chicken fillets at different core temperatures. Mean values and standard error of the mean shown.

As several consumers expressed concerns about dry chicken meat if the meat was cooked too long, we investigated how cooking loss (who will be correlating to juiciness) related to core temperatures. The effect of increasing core temperature between 55 and 70°C on increasing cooking loss is shown in Fig 7 and shows a gradual loss with higher temperature. The results are in agreement with a study on hot air and steam cooking of chicken fillets in which higher cooking temperature and longer cooking time yielded higher losses for both cooking methods [53]. It would be interesting to determine at which core temperatures consumers would perceive chicken meat as too dry, to elucidate whether there is a real conflict between safety and the preference for juicy meat.

### Evaluation of thermometers

Only one out of 75 consumers and 6.8% in the observation study and survey respectively used a food thermometer. We checked eight food thermometers (five thermometers marketed towards consumers, two thermometers primarily marketed towards professional cooks (no 6 and 7) and one tailor made laboratory thermometer (no 8)) for properties that is important for effective measurement of the core temperature of cooked chicken fillets or similar small, thin pieces of meat (Table 7).

**Table 7:** Price, probe thickness, end temperature and response time for different thermometers at 0, 70 and 100°C. Mean values and standard error for three thermometers of each type is shown. Three technical replicates were done for each item.

No.	Price Euros Sept. 2018	Probe thickness tip [mm]	Probe thickness [mm]	0°C–ice water		70°C–water bath		100°C–boiling water	
				Temperature [°C]	Response time [s]	Temperature [°C]	Response time [s]	Temperature [°C]	Response time [s]
4	5	4	4	0.3 ± 0.6	21 ± 4	64.3 ± 0.6	30 ± 9	99.3 ± 2.3	18 ± 6
5	12	3	4	0.7 ± 0.1	10 ± 4	70.7 ± 0.1	4 ± 0	100.1 ± 0.2	4 ± 1
3	15	2.5	4	0.3 ± 0.1	16 ± 4	69.1 ± 0.2	15 ± 6	99.0 ± 0.3	16 ± 4
1	19	2.5	4	-0.2 ± 0.1	6 ± 1	69.6 ± 0.3	4 ± 1	99.5 ± 0.2	3 ± 0
2	19	4	4	0 ± 0	10 ± 0	69 ± 0	14 ± 5	99 ± 0	12 ± 1
6	45	2	3.5	0.1 ± 0.0	5 ± 1	70.1 ± 0.1	4 ± 1	100.2 ± 0.2	5 ± 2
7	110	2	3.5	0.0 ± 0.1	2 ± 1	69.9 ± 0.0	2 ± 1	99.9 ± 0.1	1 ± 1
8	200	1	2 (3)	0.3 ± 0.4	2 ± 1	69.8 ± 0.3	<1	99.7 ± 0.2	<1

Most of the thermometers had features like automatic battery cut-off, switch between°C and °F and a protection of the probes. The accuracy of measuring the 3 temperatures of 0, 70 and 100°C were acceptable within +/- 1°C, except for the cheapest consumer thermometer (no. 4) who showed a temperature of 64.3°C at 70°C. The response time to be able to read stable temperatures of +/- 1°C varied substantially. Thermometers no. 2, 3 and 4 were too slow to be convenient (14–30 sec) keeping in mind that several measurements are needed to obtain be sure to measure the part of the meat with lowest obtained temperature. Acceptable response times of less than 5 seconds at 70°C were found for thermometers no. 1, 5, 6, 7 and 8. However, a very fast response of <1 to 2 seconds were found only for the two most expensive thermometers. In a US study of consumer thermometers [54], none of the test objects had a response time below 23 sec in ice water or boiling water and the authors concluded that the response time of consumer thermometers were not in accordance with the requirements. Choosing thermometers with a thin probe is reducing the loss of liquids from the meat. All consumer thermometers were similar, but with thicker probes than the thermometers intended for professionals. The price of the consumer thermometers varied between 5 and 19 Euro. Only one thermometer intended for consumers, thermometer no. 5, combined a reasonable price with low response time and good accuracy.

### Relation between food safety advice, consumer preferences and safety of cooked chicken

The results from the present study and others showed that the judging techniques for doneness recommended by food safety experts are not widely used by consumers [14]. Furthermore, no single technique, neither those used or recommended, will assure the target—inactivation of pathogens of 5 log reduction, if used alone (present study, [15, 50, 54, 55].

The use of a food thermometer to check that a safe temperature has been reached has been promoted by food safety experts for years, without much success (present work, [14]). Our survey revealed that only 6.8% of the nearly 4000 responding households across five countries indicate using a thermometer for monitoring chicken temperature during cooking. A number of barriers for uptake among consumers has been identified, of which some are linked to belief that food thermometers are not necessary and others to difficulty of selecting and using a thermometer [14]. As shown in the present study and other studies, many consumer food thermometers available on the market are too slow and there is a need for convenient, low-cost thermometers with thin probes that can be used for small pieces of meat. However, access to a good thermometer is not enough to ensure safety. Proper use of a food thermometer requires both knowledge about how to use it and some extra efforts during cooking and for maintenance. When approaching the final cooking, the probes should be inserted in multiple spots to locate the point with the lowest temperature, usually in the thickest part of the chicken fillet, and sufficient time should be allowed for reaching the actual temperature of the meat. The consumer needs to regularly calibrate the thermometer in ice water and boiling water [13]. Finally, the consumer must be aware that the probe may be contaminated during use, and it needs to be cleaned properly. The common lack of a properly functioning thermometer could be a barrier that is possible to overcome. Changing the knowledge, beliefs, skills and cooking habits of whole populations is a greater challenge.

Because of low uptake of thermometer use, colour of juices or core meat has sometimes been recommended as alternative ways of determining doneness and it is more widely used by consumers, and in particular in elderly consumers (Tables 5 and 6, [10, 48, 56]). However, the colour change of chicken meat may appear at lower temperatures than those regarded as safe (<70°C) (present work, [49]). Also, the approach is further complicated as the judgement will be highly subjective and depends on the consumers’ vision and the type of light source [57]. Furthermore, the colour of chicken depends on the raw material (breed, muscle) and product (whole meat, minced, marinated) [52, 58–61]. Therefore, colour is not a good alternative to using a thermometer as a measurement of reaching a core temperature to obtain 5 log inactivation of pathogens. Likewise, the colour of the juices will not be a proper way of measuring the heat treatment.

The development of fibrous structures in the meat due to coagulation of proteins at high temperatures seemed to correlate with reaching temperatures of 70°C. However, the texture changed gradually from about 65°C, and judging texture was not widely observed and a subtle and unarticulated method of determining if the chicken was properly cooked among consumers. In the survey, overall about 40% reported that they used inner texture to judge doneness. However, this practice was not widely shared among all countries and varied between 20 and 60%. The inner texture as a monitoring of doneness was most frequent in Romania, where the preferences for well done meat was higher than in other countries. This approach may be difficult to explain to a wide audience, but further research should focus on how consumers use inner texture to judge doneness and whether this approach is safe.

The present investigation demonstrated that it is possible to fry poultry to a core temperature of 70°C, while parts of the surface remain undercooked. About half of the consumers checked the outer colour of the chicken to see if it was cooked. The survey indicated that more people in younger age groups (>30%) check the outer colour than elderly (25.8%). For whole, intact chicken fillets, the majority of bacteria will be present on the outer surface and one could argue that the core temperature is not the most important indicator for safety [43]. If so, consumer advice should focus on proper heat treatment of the surfaces rather than the core. A challenge with using this as the only advice, is that obtaining and visually checking proper heat treatment of all surfaces of a whole chicken, chicken legs, wings or small pieces of meat is not easy. Another limitation of this strategy would be that the advice is not necessarily safe for products that are injected, as they may contain more bacteria in the interior. Moisture-enhanced products are not necessarily labelled with “injected”, but “marinated” and an indication of water content. These products are typically intended for barbeque, a situation associated with foodborne illness.

Because of large differences in the pathogen levels, consumer habits and preferences and the economic situation across the world, it is difficult to provide universal food safety messages. For example, a price of 12 euro for a food thermometer may be affordable for many Europeans, but not for the poorest part of the population in Europe or other regions of the world. Also, cooking chicken to obtain a five log reduction, which is regarded as a safe cooking process in Europe and the US, would not be sufficient to reduce the level below the virulent dose in the percentage of chicken sold in markets in Cambodia [62], which contained 10^7^−10^8^/gram *Campylobacter*.

As the last line of defence, consumers need evidence-based knowledge and convenient preparation and monitoring methods (tools or sensory) to make safe food. It is a challenge that neither is present for cooking of chicken safely. One may argue that food safety should be the response of the farmers and the food industry, but to reach a level of zero risk is so far not achievable. Food safety authorities and other risk communicators should provide science-based advice to consumers about how to mitigate risk but should also be aware of that some consumers prioritise other concerns than safety, such as taste.

## Conclusion

In conclusion, consumer practices for monitoring doneness of cooked chicken are not always safe. For example, some consumers use the inner colour of the meat or texture to judge doneness, but these approaches do not ensure that pathogens are inactivated. Many consumers were concerned about juiciness of chicken, and safety concerns may have lower priority than taste. It is worrying that the advice on chicken cooking from the authorities or organisations working with food safety communication towards consumers are not always safe or likely to be adopted by consumers. For example, the use of food thermometers to measure the core temperature is often recommended, but this approach is not only difficult to apply in practice, it is also not safe as bacteria may survive on the surface even at proper core temperatures. To develop safer and more adoptable consumer advice, a risk analysis based on data covering the level of pathogens in several raw chicken products and from several preparation methods, combined with various consumer practices should be conducted.

Food safety messages towards European consumers should be built risk reduction potential, but also take into account present consumer practices and preferences to obtain adoption. For the moment, the main focus should be on proper heat treatment of all surfaces (frying all meat surfaces or cooking in sauce). A combination of judgement of the colour (pale for chicken fillets) and development of fibrous structure in the thickest part of the chicken meat should also be recommended.

## Supporting information

S1 Fig Overview of the research participants. The number of informants in different categories is shown. One participant did not provide information about education. Three participants did not inform about their income.
(TIF)

S1 Table Socio-demographic characteristics of the surveyed households. (DOCX)

S2 Table Self-reported practices per age group, all countries. (DOCX)
