# Supplementary figures and images for: Cooking chicken at home: Common or recommended approaches to judge doneness may not assure sufficient inactivation of pathogens
Source: PLoS One. 2020 Apr 29;15(4):e0230928. doi: 10.1371/journal.pone.0230928 (PMC7313536; doi:10.1371/journal.pone.0230928)

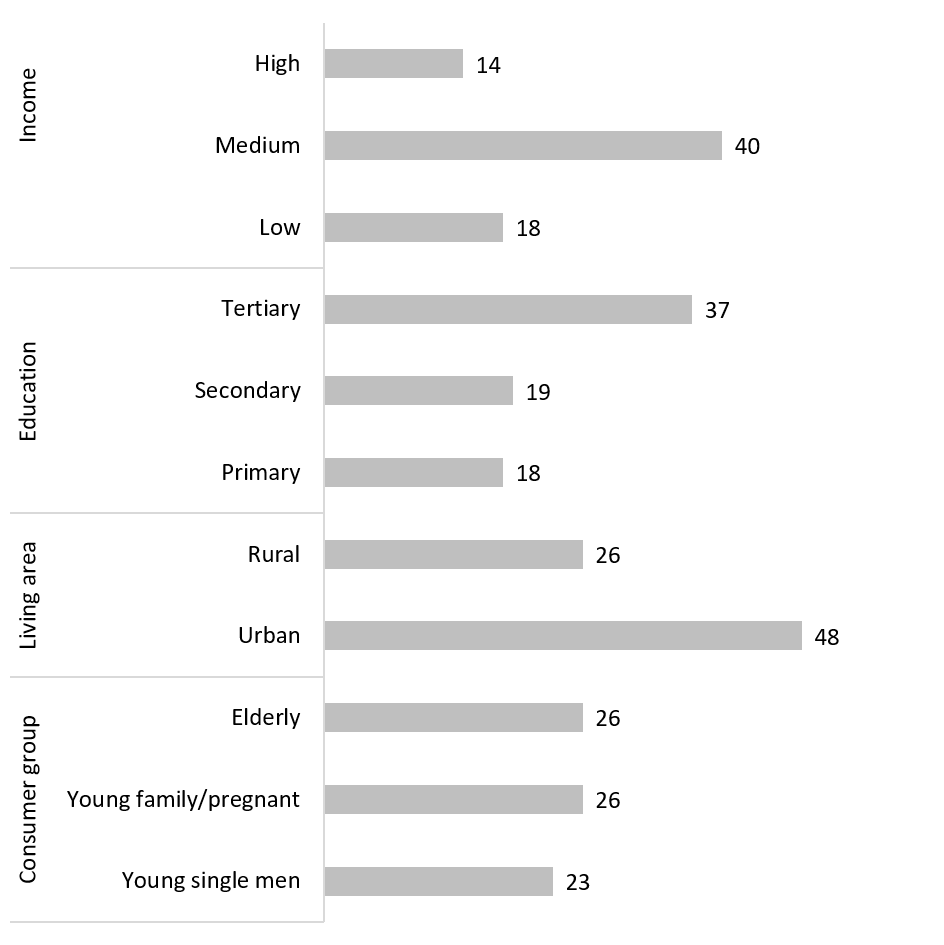

Supplement: S1 Fig — The number of informants in different categories is shown. One participant did not provide information about education. Three participants did not inform about their income. (TIF) [file pone.0230928.s001.tif]
