# Supplementary material for: Cooking chicken at home: Common or recommended approaches to judge doneness may not assure sufficient inactivation of pathogens
Source: PLoS One. 2020 Apr 29;15(4):e0230928. doi: 10.1371/journal.pone.0230928 (PMC7313536; doi:10.1371/journal.pone.0230928)
Supplement: S1 Table — (DOCX) [file pone.0230928.s002.docx]

Table S1 Socio-demographic characteristics of the surveyed households

|  | **FRANCE**  **(n=706)** | **NORWAY**  **(n=844)** | **PORTUGAL**  **(n=609)** | **ROMANIA**  **(n=894)** | **UK**  **(n=916)** | **POOLED**  **(n=3969)** |
| --- | --- | --- | --- | --- | --- | --- |
| Household mean size (SD) | 2.6 (1.3) | 2.4 (1.3) | 2.9 (1.2) | 2.9 (1.2) | 2.7 (1.3) | 2.7 (1.3) |
| Household type (%) |  |  |  |  |  |  |
| *Without children* | 60.0 | 67.3 | 50.0 | 52.1 | 63.4 | 59.0 |
| *With children* | 40.0 | 32.7 | 50.0 | 47.9 | 36.6 | 41.0 |
| Male respondents (%)  Age, in years | 48.9 | 49.9 | 53.9 | 50.3 | 50.2 | 50.5 |
| *Min-Max* | 16-83 | 16-85 | 16-81 | 16-82 | 16-90 | 16-90 |
| *Mean* | 45.9 | 44.9 | 45.9 | 45.1 | 46.7 | 45.7 |
| Education (%) |  |  |  |  |  |  |
| *Low*  *Medium* | 8.4  32.4 | 10.0  42.3 | 12.5  38.1 | 1.0  31.7 | 3.3  40.1 | 6.5  37.0 |
| *High* | 58.6 | 43.4 | 48.9 | 66.9 | 55.8 | 55.1 |
| Households with at least one risk group* (%) | 58.4 | 48.8 | 53.9 | 60.2 | 55.6 | 55.4 |

*Pregnancy, children under six years old, teenagers and young adults (16-25 years) leaving alone, men 25-30 years old leaving alone, diabetes, immunodeficiency, or above 65 years old.
