# Supplementary material for: Cooking chicken at home: Common or recommended approaches to judge doneness may not assure sufficient inactivation of pathogens
Source: PLoS One. 2020 Apr 29;15(4):e0230928. doi: 10.1371/journal.pone.0230928 (PMC7313536; doi:10.1371/journal.pone.0230928)
Supplement: S2 Table — (DOCX) [file pone.0230928.s003.docx]

Table S2 Self-reported practices per age group, all countries

| **Age** |  | **16-30**  **(n=949)** | **31-45**  **(n=1089)** | **46-60**  **(n=965)** | **61-75**  **(n=913)** | **76-90**  **(n=53)** | **All ages**  **(n=3969)** |
| --- | --- | --- | --- | --- | --- | --- | --- |
| **Doneness of chicken (%)** |  | 10.0 | 7.9 | 6.7 | 5.8 | 4.9 | 7.6 |
| *Less done* |  | 2.0^C^ | 1.2^BC^ | 0.3^AB^ | 0.1^A^ | 0.0^A^ | 0.9^a^ |
| *Medium-well* |  | 20.1^B^ | 16.5^B^ | 17.7^B^ | 18.1^B^ | 13.2^A^ | 18.0^b^ |
| *Well-done* |  | 51.8^B^ | 58.3^C^ | 58.5^BC^ | 58.5^B^ | 62.3^A^ | 56.9^d^ |
| *Very well done* |  | 26.0^B^ | 24.0^B^ | 23.4^B^ | 23.3^B^ | 24.5^A^ | 24.2^c^ |
| **Strategy for checking doneness (%)** |  |  |  |  |  |  |  |
| *Outside colour*  *Inside colour*  *Inside texture*  *Juices*  *Thermometer*  *Time* |  | 33.3^c^  49.0^d^  33.1^c^  15.2^b^  8.5^a^  14.4^b^ | 31.1^c^  48.7^d^  34.1^c^  17.3^b^  6.7^a^  15.9^b^ | 31.3^c^  54.1^e^  43.8^d^  19.9^b^  5.8^a^  14.8^b^ | 26.8^c^  47.4^d^  46.8^d^  23.5^c^  6.0^a^  16.4^b^ | 22.6^ab^  35.8^ab^  35.8^b^  34.0^ab^  7.5^a^  22.6^ab^ | 30.6^d^  49.6^f^  39.2^e^  19.1^c^  6.8^a^  15.5^b^ |

*Expressed in days/month equivalents

^A, B, C, D^: Different letters indicate significant differences across age groups (Cochran’s Q test, 5% level)

^a,b, c, d, e, f^: Different letters indicate significant differences across alternatives within age groups (Cochran’s Q test, 5% level)
